# The Association Between Healthy Lifestyle Score Trajectory and Frailty in Middle-Aged and Older Adults in Korea: Findings from the Korean Longitudinal Study of Aging (2006–2024)

**DOI:** 10.3390/medicina62040766

**Published:** 2026-04-15

**Authors:** Young Long Choi, Bon Hee Gu, Jeong Min Yang

**Affiliations:** 1 Bio-Active Product Research Center, Dankook University, Cheonan 31116, Republic of Korea; younglong@dankook.ac.kr; 2 Biohealth Convergence Innovation University Project, Dankook University, Cheonan 31116, Republic of Korea; 9qhsgml@dankook.ac.kr; 3 Office of Public Healthcare, National Medical Center, Seoul 04564, Republic of Korea

**Keywords:** frailty, healthy lifestyle, trajectory modeling, sex differences, Korean Longitudinal Study of Aging, older adults

## Abstract

*Background and Objectives*: represents a major public health challenge in rapidly aging societies. While lifestyle behaviors are established modifiable risk factors for frailty, the longitudinal impact of composite lifestyle trajectories—particularly by sex—remains poorly understood. This study examined sex-stratified associations between Healthy Lifestyle Score Trajectories (HLSTs) and frailty among community-dwelling middle-aged and older adults in South Korea. Using 19 years of nationally representative panel data from the Korean Longitudinal Study of Aging (2006–2024), we analyzed 6603 participants (2684 males; 3919 females). *Materials and Methods*: Group-Based Trajectory Modeling was applied to Waves 1–6 to derive sex-specific HLSTs based on smoking, alcohol consumption, physical activity, and body mass index. Generalized Estimating Equations were used to assess longitudinal associations between HLSTs and Frailty Index (FI) scores across Waves 6–10, adjusting for sociodemographic covariates. *Results*: Five distinct HLSTs were identified in both sexes. In both males and females, persistently poor or deteriorating trajectories were independently associated with higher FI scores relative to the Favorable HLST reference group. The effect size for Poor HLST was more than twice as large in females (B = 0.039) than in males (B = 0.018), consistent with the sex-frailty paradox. Among females, the Improving HLST group did not demonstrate a statistically significant frailty benefit (B = 0.014, *p* = 0.091). Stratified analyses revealed that the lifestyle–frailty association among males was significant only in rural-dwelling participants, whereas in females the association was consistent across both urban and rural settings. *Conclusions*: Persistently unfavorable composite lifestyle trajectories were independently associated with higher frailty burden, with disproportionately greater impact in women. Late-life lifestyle improvement was not significantly associated with reduced frailty in women, reinforcing the importance of early and sustained behavioral maintenance. The rural-specific association in men highlights the role of structural disadvantage in amplifying lifestyle-related frailty risk. However, given the observational design of this study, the possibility of reverse causality cannot be excluded, and these findings should be interpreted as associative rather than causal. These findings support sex-sensitive, trajectory-based, and geographically tailored frailty prevention strategies.

## 1. Introduction

Frailty is a clinically recognized geriatric syndrome characterized by the progressive accumulation of age-related deficits across physiological, functional, and psychosocial domains, resulting in diminished homeostatic reserve and increased vulnerability to adverse health outcomes [1,2]. As a dynamic and potentially reversible state, frailty has been associated with elevated risks of disability, hospitalization, cognitive decline, and mortality in older adults, representing a major public health concern worldwide [3,4]. With global population aging accelerating at an unprecedented pace—older adults aged 65 years and over are projected to account for approximately 16% of the world’s population by 2050—understanding the modifiable determinants of frailty has become a research priority of increasing urgency [5].

South Korea presents a particularly compelling context for the study of frailty. The country has undergone one of the most rapid demographic transitions in recorded history, with the proportion of adults aged 65 and older rising from 7% in 2000 to over 18% in 2023, and projected to exceed 40% by 2060 [6]. This accelerated aging trajectory is accompanied by a growing burden of frailty-related morbidity and functional dependency, placing substantial strain on the healthcare system. Against this backdrop, identifying upstream determinants and risk factors for frailty among middle-aged and older Korean adults is essential for the development of effective preventive strategies.

Among modifiable risk factors, lifestyle behaviors—including physical inactivity, unhealthy dietary patterns, tobacco use, and harmful alcohol consumption—have been consistently implicated in the pathogenesis of frailty [7,8,9]. These behaviors often co-occur and interact synergistically, suggesting that a composite measure of lifestyle health may offer greater epidemiological utility than evaluating individual behaviors in isolation [10]. The Healthy Lifestyle Score (HLS), an aggregate index reflecting adherence to multiple healthy behaviors, has been proposed as a parsimonious tool for capturing the cumulative impact of lifestyle on health outcomes [11]. Importantly, lifestyle behaviors are not static; they evolve over time in response to aging, life transitions, and societal changes. Conventional cross-sectional or single time-point assessments fail to capture this dynamic nature, potentially obscuring critical associations between lifestyle trajectories and health outcomes.

To address this limitation, Group-Based Trajectory Modeling (GBTM) has emerged as a powerful analytical framework for identifying distinct latent subgroups that follow heterogeneous developmental patterns over time [12]. Applied to longitudinal lifestyle data, GBTM enables the identification of Healthy Lifestyle Score Trajectories (HLSTs)—subgroup-specific patterns of lifestyle change over time—that may differentially predict frailty outcomes. Prior studies have applied trajectory modeling to individual health behaviors such as physical activity and smoking, yet investigations into composite lifestyle trajectories and their association with frailty remain scarce, particularly in Asian populations.

Moreover, growing evidence suggests that the relationship between lifestyle behaviors and frailty may be substantially modified by sex. Men and women differ markedly in the prevalence, patterning, and biological consequences of lifestyle risk factors, as well as in the underlying mechanisms of frailty [13,14]. For instance, women exhibit higher rates of frailty despite lower mortality compared to men—a phenomenon termed the ‘frailty-survival paradox’—which may be partly attributable to sex-specific differences in behavioral trajectories and their downstream effects on functional reserve [15]. Despite this recognition, few longitudinal studies have explicitly examined sex-stratified associations between composite lifestyle trajectories and frailty using population-representative data.

This study aimed to address these gaps by examining the association between sex-stratified HLSTs and frailty, as measured by the Frailty Index (FI), among community-dwelling middle-aged and older adults in South Korea. Using data from the Korean Longitudinal Study of Aging (KLoSA)—a nationally representative biennial panel survey initiated in 2006 and followed through 2024—we applied GBTM to Waves 1–6 data to identify sex-specific HLSTs based on four behavioral domains: smoking, alcohol consumption, physical activity, and body mass index. We then employed Generalized Estimating Equations (GEE) to evaluate the longitudinal associations between these trajectory groups and FI scores derived from Waves 6–10, while adjusting for relevant sociodemographic and health-related confounders.

The findings from this study carry important implications for the design of sex-tailored public health interventions targeting frailty prevention in aging populations. By characterizing how distinct patterns of lifestyle change over time relate to frailty outcomes differently in men and women, this research contributes novel evidence to the growing body of literature on the modifiable determinants of healthy aging in East Asia.

## 2. Materials and Methods

This study utilized data from the KLoSA collected between 2006 and 2024 to construct separate lifestyle trajectory models for male and female groups. KLoSA is a panel survey that repeatedly tracks the same respondents over time using identical questionnaires, offering the advantages of both cross-sectional and longitudinal data. All variables were measured repeatedly from Wave 1 to Wave 6, allowing for the collection of observations across multiple time points. The survey was designed to represent community-dwelling individuals aged 45 and older across South Korea, using a multistage stratified probability sampling method based on region and housing type. The sample was drawn by the Korea Employment Information Service, and in cases where selected individuals declined to participate, substitute participants were recruited from a pre-sampled reserve within the same area.

In the first wave of the survey conducted in 2006, a total of 10,254 individuals from 6171 households (with an average of 1.7 individuals per household) participated. In the tenth wave conducted in 2024, a total of 7000 respondents including 738 new participants completed the follow-up survey, accounting for 78.1% of the original panel (6592 individuals).

To analyze the association between HLST and Frailty, 4892 newly enrolled participants from Waves 5 to 10 were excluded from the initial KLoSA sample of 15,146 individuals. Next, 251 participants with missing data on smoking, alcohol consumption, BMI, or PA during Waves 1 to 6 were excluded, along with 1664 individuals who died prior to the 2016 baseline. An additional 1736 participants with missing values for dependent or control variables in Waves 6 to 10 were also excluded. Ultimately, a total of 6603 individuals (2684 males and 3919 females) who participated in all ten waves from Wave 1 to Wave 10 were included in the final analysis. The sample selection process is illustrated in Supplementary Table S1.

To assess the potential impact of sample attrition, baseline characteristics were compared between included and excluded participants, as presented in Supplementary Table S2. Statistically significant differences were observed in age, income level, and social activity, suggesting that the excluded participants were not entirely comparable to the analytic sample, and that these differences should be considered when interpreting the generalizability of the findings.

### 2.1. Independent Variables

In this study, an HLST was calculated by assigning scores to each lifestyle indicator [16]. First, smoking status was categorized as follows: non-smokers (two points), past smokers (one point), and current smokers (zero points). Second, alcohol consumption was classified as: non-drinkers (two points), past drinkers (one point), and current drinkers (zero points). Third, physical activity (PA) was classified by weekly duration as follows: ≥150 min (two points), <150 min (one point), and none (zero points). Lastly, BMI was evaluated according to the WHO Asia-Pacific classification: normal weight (18.6~22.9 kg/m^2^, 2 points), borderline overweight (23.0~24.9 kg/m^2^, one point), and underweight (≤18.5 kg/m^2^, zero point) or obesity (≥25.0 kg/m^2^, zero points). The total score of the lifestyle indicators ranged from 0 to 8, with higher scores indicating healthier lifestyle behaviors. HLST were evaluated using a Group-Based Trajectory Model (GBTM) based on the continuous HLS variable, and separate trajectories were derived for males and females. Model fit for the sex-stratified HLST trajectories was assessed using the Akaike Information Criterion (AIC), Bayesian Information Criterion (BIC), and Average Posterior Probability (APP), which are presented in Supplementary Tables S3 and S4 for males and females, respectively.

### 2.2. Dependent Variables

The dependent variable was frailty, assessed using the FI. The FI is a multidimensional indicator of health status that captures the accumulation of deficits across various physiological and functional domains associated with aging. It is computed as the ratio of observed health deficits to the total number of considered variables and has been widely validated and applied in frailty research internationally [17]. Variables were selected based on previous studies that calculated frailty indices using data from the KLoSA [18]. The FI was measured using 34 variables across six domains: self-rated health, physical condition, activities of daily living, instrumental activities of daily living, chronic conditions, and mental status. Each variable was scored on a scale ranging from 0 to 1, with higher scores indicating more severe frailty. Physical frailty included the domains of self-rated health, physical condition, activities of daily living, instrumental activities of daily living, and chronic conditions. The grip strength of both hands was measured twice using a dynamometer, and the overall average of all measurements was used. The cutoff scores for handgrip strength were 28.6 for men and 16.4 for women. This criterion was proposed in previous research to more accurately reflect the characteristics of the Korean older adult population and has since been adopted in studies [19]. Scores at or below the cutoff were assigned a value of 1 point, whereas scores above the cutoff were assigned 0 points. Psychological frailty was measured using four items from the 10-item version of the Center for Epidemiological Studies Depression (CES-D) Scale. Among these items, the question “I had trouble keeping my mind on what I was doing” was replaced with “I felt sad during the past week” from the fifth wave of the survey. In addition, regarding chronic diseases, the incontinence item was only surveyed among female participants. Therefore, this item was replaced with a corresponding item for prostate disease for male participants. The FI measurement criteria used in this study are presented in Supplementary Table S5.

### 2.3. Control Variables

Age was divided into four groups: 55–64, 65–74, and 75 years or older. ‘Educational level’ was classified into four categories: elementary school or less, middle school, high school, and college or higher. ‘Residential region’ was categorized into urban areas (including Seoul, Daejeon, Daegu, Busan, Incheon, Gwangju, and Ulsan) and rural areas. ‘Income level’ was divided into four groups: low, lower-middle, upper-middle, and high income. ‘Marital status’ was classified into two groups: married and unmarried. ‘Health Insurance Status’ was divided into two groups: Medical aid and National Health Insurance. Finally, ‘Economic activity’ and ‘Social activity’ were classified into two groups, respectively: No and Yes.

### 2.4. Analytical Approach and Statistics

In this study, to examine the impact of HLST on frailty among middle-aged and older Korean adults, descriptive statistics were conducted to assess group differences in baseline characteristics. GBTM was employed to identify lifestyle patterns, and a GEE model was used to analyze the association between HLST and frailty (See Supplementary Table S6).

First, to derive HLST based on smoking, alcohol consumption, BMI, and PA, a GBTM was applied using Waves 1–6 of the KLoSA dataset. The trajectory model is a method used to estimate unobserved subgroups and differences between groups within specified constraints, based on observed data. Subsequently, to analyze the association between lifestyle trajectory groups and frailty, a GEE model was employed using panel data from Waves 6–10, considering the repeated measures structure of the FI. The analysis was adjusted for potential confounders.

The GEE model was specified with an identity link function and a normal distribution, given that the frailty index was treated as a continuous outcome variable. A compound symmetry (exchangeable) correlation structure was adopted to account for the within-subject correlation across repeated measurements, under the assumption that the correlation between any two time points was constant regardless of the interval between waves. This specification was deemed appropriate given the longitudinal nature of the KLoSA data, in which repeated observations were nested within individuals across multiple survey waves. Subject-level clustering was defined by individual participant identifiers, and wave was included as a covariate to account for time effects across the follow-up period.

Data management and statistical analyses were performed using SAS Enterprise Guide 8.3 (SAS Institute Inc., Cary, NC, USA) and R Studio 4.3.0 (R Studio Inc., Boston, MA, USA). *p*-value of <0.05 was considered statistically significant.

## 3. Results

### 3.1. General Characteristics of Study Participants

A total of 6603 participants were included in the final analysis, comprising 2684 males and 3919 females. At baseline, the mean FI was 0.153 (SD = 0.108) among males and 0.176 (SD = 0.122) among females, indicating a higher overall frailty burden in women. The general characteristics of the study population, stratified by sex and HLST group, are presented in Table 1.

### 3.2. Healthy Lifestyle Score Trajectories by Sex

Among males, five distinct HLSTs were identified: Poor HLST (11.1%), Moderate HLST (45.4%), Severely Deteriorating HLST (4.2%), Mildly Deteriorating HLST (6.6%), and Favorable HLST (32.7%). Significant differences in baseline FI were observed across these trajectory groups (*p* < 0.0001), with the Poor HLST group recording the highest mean FI (0.163) and the Favorable HLST group the lowest (0.148).

**Figure 1:**
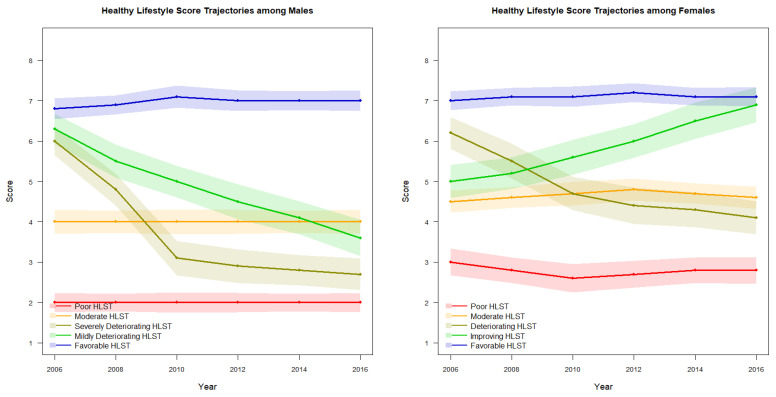
Trajectory of Healthy Lifestyle Score by sex.

Among females, five HLSTs were identified: Poor HLST (4.8%), Moderate HLST (33.8%), Deteriorating HLST (13.8%), Improving HLST (4.0%), and Favorable HLST (43.6%). Significant FI differences were also observed across female trajectory groups (*p* < 0.0001), with Poor HLST yielding the highest mean FI (0.203) and Favorable HLST the lowest (0.158). Trajectory patterns are illustrated in Figure 1.

### 3.3. Association Between HLST and Frailty Index

Table 2 presents the adjusted associations between HLST and FI from GEE analyses. Among males, three trajectory groups were significantly associated with higher FI relative to the Favorable HLST reference: Poor HLST (B = 0.018, 95% CI: 0.007–0.029, *p* = 0.002), Moderate HLST (B = 0.008, 95% CI: 0.000–0.016, *p* = 0.042), and Severely Deteriorating HLST (B = 0.019, 95% CI: 0.005–0.033, *p* = 0.010). The Mildly Deteriorating HLST group did not reach statistical significance (*p* = 0.181).

Among females, Poor HLST (B = 0.039, 95% CI: 0.024–0.055, *p* < 0.0001), Moderate HLST (B = 0.022, 95% CI: 0.016–0.028, *p* < 0.0001), and Deteriorating HLST (B = 0.019, 95% CI: 0.010–0.027, *p* < 0.0001) were all significantly associated with higher FI compared to the Favorable HLST reference. The Improving HLST group was not statistically significant (*p* = 0.091). Notably, the effect size for Poor HLST was more than twice as large in females (B = 0.039) than in males (B = 0.018), suggesting a stronger frailty impact of persistently poor lifestyle behaviors among women.

### 3.4. Stratified Analysis by Sex and Region

Table 3 presents the stratified analyses by residential region. Among urban-dwelling males, no HLST group showed a statistically significant association with FI. However, among rural-dwelling males, Poor HLST (B = 0.029, 95% CI: 0.013–0.044, *p* < 0.001) and Severely Deteriorating HLST (B = 0.026, 95% CI: 0.005–0.047, *p* = 0.016) were significantly associated with higher FI, with Mildly Deteriorating HLST also reaching borderline significance (B = 0.018, *p* = 0.050).

Among females, unfavorable lifestyle trajectories were significantly associated with higher FI regardless of residential region. In urban areas, Poor HLST (B = 0.045, *p* < 0.0001), Moderate HLST (B = 0.023, *p* < 0.0001), and Deteriorating HLST (B = 0.025, *p* < 0.0001) were all significant. In rural areas, similar patterns were observed for Poor HLST (B = 0.037, *p* = 0.001), Moderate HLST (B = 0.022, *p* < 0.0001), and Deteriorating HLST (B = 0.015, *p* = 0.009). These findings indicate that the association between unfavorable lifestyle trajectories and frailty was confined to rural areas in males, whereas in females it was consistent across both urban and rural settings.

## 4. Discussion

This study investigated the sex-stratified associations between HLST and frailty, as measured by the FI, among community-dwelling middle-aged and older adults in South Korea. The principal findings of this study are fourfold. First, persistently poor or deteriorating lifestyle trajectories were independently associated with higher FI scores in both sexes, even after comprehensive covariate adjustment. Second, the frailty impact of unfavorable lifestyle trajectories was substantially more pronounced in women than in men, as evidenced by an effect size more than twice as large in females. Third, among women, classification into the Improving HLST group did not confer a statistically significant frailty benefit relative to the Favorable reference group. Fourth, the association between unfavorable HLSTs and frailty among men was significant only in rural-dwelling participants, whereas in women, the association was consistent across both urban and rural settings. Each of these findings is discussed in turn below.

The independent association between persistently poor or deteriorating HLSTs and higher FI scores extends prior cross-sectional evidence by demonstrating that the frailty-relevant burden of lifestyle behavior reflects the cumulative longitudinal patterning of health behaviors, rather than any single time-point assessment. This interpretation is broadly consistent with a growing body of trajectory-based longitudinal evidence. Wilhelmsen et al. [10], using data from a Finnish birth cohort followed over 17 years, reported that cessation of regular exercise and newly acquired sleep disturbance were independently associated with a faster rate of frailty progression from late midlife into older age. McPhee et al. [20], drawing on 10-year ELSA data from 8649 non-frail adults, demonstrated that vigorous physical activity significantly attenuated frailty trajectories across all age groups. At the composite lifestyle level, Yue et al. [21], using CHARLS data spanning nine years, found that a persistently low HLST was independently associated with incident functional disability, corroborating the trajectory-frailty link observed in the present study. Jung et al. [19], utilizing KLoSA data from 2006 to 2022—a dataset closely paralleling the present study—further demonstrated that persistent physical inactivity was significantly associated with higher frailty in both sexes among Korean middle-aged and older adults. Furthermore, evidence from the NHANES-based study [22] showed that participants with an optimal composite healthy lifestyle had a 39% lower risk of frailty compared with those with a poor lifestyle, reinforcing the protective value of sustained multi-domain behavioral adherence. Taken together, these convergent findings underscore the need to move beyond static behavioral assessments and adopt longitudinal, trajectory-based profiling as a cornerstone of frailty prevention strategy.

The substantially larger association between Poor HLST and FIS in females (B = 0.039) compared with males (B = 0.018) is consistent with the well-established ‘frailty-survival paradox,’ whereby women exhibit higher frailty prevalence and severity than men of equivalent age despite lower mortality [13,14]. Gordon et al. [14], in a systematic review and meta-analysis of seven large community cohort studies encompassing 37,426 participants, confirmed that females had significantly higher FI scores than males across all five-year age groups. Several mechanisms may account for the amplified frailty impact of poor lifestyle trajectories observed specifically in women. Biologically, women face steeper age-related declines in musculoskeletal reserve, hormonal transitions at menopause, and greater susceptibility to chronic low-grade inflammation—factors that may heighten the frailty-promoting effects of sustained adverse behavioral exposures [23,24]. From a behavioral and structural standpoint, the present data reveal that women in the analytic sample were markedly more likely to be economically inactive (73.5% vs. 49.1% in men) and to have lower rates of social activity participation, both of which are independently implicated in frailty accumulation [8]. Ko and Choi [25], using KLoSA data, similarly identified regular exercise and socioeconomic status as among the most protective factors against frailty, specifically among Korean older women. The intersection of biological vulnerability and sustained behavioral and sociostructural disadvantage may render women disproportionately sensitive to the frailty-promoting effects of poor lifestyle trajectories, reinforcing the imperative for sex-sensitive prevention frameworks.

The absence of a statistically significant frailty benefit in women classified into the Improving HLST group (B = 0.014, *p* = 0.091) is noteworthy and warrants careful interpretation. One plausible explanation is a ‘lag effect’: behavioral improvements initiated in mid-to-late life may require a longer observation window to manifest as measurable reductions in deficit accumulation, given that the FI reflects the cumulative biological residue of decades of adverse exposure [2,26]. This interpretation is supported by Huang et al. [27], who found in a Chinese elderly cohort that improving lifestyle from unhealthy to moderate over three years was associated with an attenuated frailty risk (HR = 0.83, 95% CI: 0.70–0.99) relative to consistently healthy patterns (HR = 0.64, 95% CI: 0.54–0.75), suggesting diminishing protective returns when behavioral change is initiated late. An alternative explanation involves reverse causality: individuals who improved their lifestyle in later life may have done so in response to early health deterioration, creating a selection effect that attenuates the apparent benefit of behavioral improvement. Regardless of mechanism, these findings support the life-course principle that the protective effects of healthy behavioral patterns are most robust when initiated and maintained early—prior to the consolidation of frailty-promoting deficit trajectories [28]—and have direct implications for the timing and targeting of public health interventions.

The finding that the lifestyle–frailty association among men was statistically significant only in rural-dwelling participants resonates with Korean-specific evidence on rural-urban frailty disparities. Jang et al. [29] reported that rural-dwelling older Koreans had markedly higher frailty prevalence (17.4%) compared with their urban counterparts (10.3%), with muscle weakness and exhaustion disproportionately prevalent in rural settings. Using Korean Frailty and Aging Cohort Study data from 2593 community-dwelling older adults, Seo et al. [30] further demonstrated that limited access to recreational facilities and poor neighborhood aesthetics were significantly associated with frailty, specifically among rural residents. The amplified lifestyle–frailty association among rural men likely reflects a compounding effect: suboptimal behavioral trajectories interact with structural barriers—including reduced healthcare accessibility, limited physical activity infrastructure, and greater socioeconomic vulnerability—to potentiate frailty risk beyond what lifestyle factors alone would predict. In contrast, urban-dwelling men may benefit from a denser service environment and richer social infrastructure that partially buffers the frailty impact of adverse behavioral patterns. For women, the lifestyle–frailty association was significant and consistent across both residential settings, suggesting that women’s heightened biological susceptibility is not attenuated by the structural advantages of urban residence—a finding that distinguishes the female frailty-lifestyle relationship from its male counterpart.

The findings of this study carry several important implications for public health policy and clinical practice. First, the consistent independent association between unfavorable HLSTs and higher frailty burden—demonstrated across both sexes and after adjustment for comprehensive confounders—provides robust population-level evidence that composite lifestyle trajectories represent meaningful, modifiable targets for frailty prevention in aging populations. Frailty prevention programs should incorporate trajectory-based behavioral monitoring, enabling early identification of individuals whose lifestyle patterns are deteriorating before clinical frailty manifests. Second, the substantially greater frailty impact of poor lifestyle trajectories among women necessitates sex-differentiated intervention strategies. Programs specifically designed to address the economic inactivity, social isolation, and biological vulnerabilities characteristic of aging Korean women merit prioritization. Third, the null finding for the Improving HLST group suggests that late-life behavioral correction is insufficient to reverse accumulated frailty deficits; accordingly, public health messaging should emphasize the early adoption and sustained maintenance of multi-domain healthy behaviors rather than focusing solely on late-life improvement. Fourth, the rural-specific significance of the lifestyle–frailty association among men highlights the need for geographically tailored frailty prevention initiatives that address the structural determinants of healthy aging in rural communities, including expanded access to physical activity facilities, community health centers, and integrated health services.

This study possesses several notable strengths. The use of KLoSA spanning 19 years provides exceptional temporal depth with a large, nationally representative sample (n = 6603). The application of GBTM with sex-stratified trajectory derivation captures behavioral heterogeneity that conventional mean-trajectory approaches would obscure, and the 34-item FI affords a sensitive, multidimensional frailty outcome. The GEE framework appropriately accounts for the repeated-measures structure of panel data, yielding robust population-averaged estimates.

Nevertheless, several limitations must be acknowledged. All lifestyle variables were self-reported, introducing potential recall and social desirability bias; objective measures such as accelerometry or biomarkers were unavailable within the KLoSA framework. To mitigate this limitation, each lifestyle behavioral domain was operationalized using validated classification indices informed by a thorough review of the prior literature, ensuring that the categorization of lifestyle variables was grounded in well-established and reproducible criteria. The HLS was restricted to four behavioral domains, excluding dietary quality, sleep duration, and sedentary time—each with established relevance to frailty—potentially underestimating the full explanatory scope of the trajectory classification. Restriction to participants completing all ten survey waves may have introduced survivorship bias, likely resulting in an underestimation of the true lifestyle–frailty association among the most vulnerable subgroups. To address this concern, baseline characteristics were systematically compared between included and excluded participants, revealing significant differences in age, income level, and social activity; these findings are presented in Supplementary Table S2 and should be considered when interpreting the generalizability of the present findings. Furthermore, while the GEE model adjusted for a broad range of covariates, unmeasured confounders including genetic predisposition and inflammatory biomarkers may account for residual associations. Additionally, although BMI was incorporated as a modifiable lifestyle behavioral domain in the HLS, consistent with prior composite lifestyle scoring approaches in older adult populations, the potential conceptual overlap between BMI as an exposure component and its possible contribution to frailty-related deficits in the outcome warrants acknowledgment. Moreover, the binary scoring of both underweight and obesity as equivalent unfavorable states may not fully capture the nonlinear and directionally distinct relationships between these BMI categories and frailty in older adults. The possibility of reverse causality also warrants consideration; individuals exhibiting improving lifestyle trajectories may have modified their behaviors in response to early or subclinical frailty symptoms rather than as an antecedent behavioral change, thereby obscuring the true directionality of the lifestyle–frailty association. Future studies employing stricter temporal precedence criteria or instrumental variable approaches would be better positioned to clarify the causal relationship between lifestyle trajectory patterns and frailty outcomes. Additionally, although the GBTM-derived trajectory classification is data-driven, the possibility of within-group heterogeneity in the Improving HLST group cannot be entirely excluded. Variability in the magnitude of lifestyle improvement, the sustainability of behavioral change, and the timing of improvement relative to frailty assessment may have obscured differential associations with frailty outcomes, and the null finding observed in this group should therefore not be interpreted as definitive evidence of the absence of benefit from lifestyle improvement.

## 5. Conclusions

This study provides longitudinal evidence that persistently poor or deteriorating composite lifestyle trajectories are independently associated with higher frailty burden among community-dwelling middle-aged and older adults in South Korea, after adjustment for comprehensive sociodemographic covariates. However, given the observational design of the present study, the possibility of reverse causality cannot be entirely excluded; frail individuals may have progressively adopted poorer lifestyle behaviors as a consequence of declining health, rather than as an antecedent cause, and these findings should therefore be interpreted as associative rather than causal. The frailty impact of unfavorable lifestyle trajectories was substantially more pronounced in women than in men, consistent with the sex-frailty paradox and indicative of women’s heightened biological and structural vulnerability to sustained behavioral risk. Among women, lifestyle improvement initiated in later life was not significantly associated with a reduction in frailty, reinforcing the life-course principle that early adoption and sustained maintenance of healthy behaviors are essential for frailty prevention. In men, the lifestyle–frailty association was significant only among rural-dwelling participants, suggesting that structural and environmental disadvantage amplifies the frailty-promoting effects of poor behavioral trajectories. Collectively, these findings call for sex-sensitive, trajectory-based, and geographically tailored frailty prevention strategies, and underscore the importance of longitudinal behavioral monitoring as a cornerstone of healthy aging policy in rapidly aging populations.

## Figures and Tables

**Table 1:** General characteristics of subjects included for analysis at baseline.

Variables	Total		Male		*p*-Value (Effect Size)	Total		Female		*p*-Value (Effect Size)
			FIS					FIS		
	N	(%)	Mean	SD		N	(%)	Mean	SD	
Total	2684	(100.0)	0.153	(0.108)		3919	(100.0)	0.176	(0.122)	
**HLST among males**					<0.0001					
Poor HLST	297	(11.1)	0.163	(0.125)	(0.0018)	N/A				
Moderate HLST	1218	(45.4)	0.151	(0.107)						
Severely Deteriorating HLST	112	(4.2)	0.151	(0.091)						
Mildly Deteriorating HLST	178	(6.6)	0.154	(0.110)						
Favorable HLST	879	(32.7)	0.148	(0.091)						
**HLST among females**										<0.0001
Poor HLST			N/A			190	(4.8)	0.203	(0.123)	(0.0186)
Moderate HLST						1324	(33.8)	0.192	(0.124)	
Deteriorating HLST						542	(13.8)	0.183	(0.123)	
Improving HLST						155	(4.0)	0.162	(0.129)	
Favorable HLST						1708	(43.6)	0.158	(0.117)	
**Age**					<0.0001					<0.0001
55–64	984	(36.7)	0.108	(0.074)	(0.1616)	1373	(35.0)	0.109	(0.070)	(0.2398)
65–74	900	(33.5)	0.148	(0.094)		1209	(30.8)	0.167	(0.094)	
≥75	800	(29.8)	0.215	(0.128)		1337	(34.1)	0.252	(0.142)	
**Region**					0.820					0.867
Urban	1143	(42.6)	0.151	(0.110)	(0.0369)	1691	(43.1)	0.170	(0.119)	(0.0821)
Rural	1541	(57.4)	0.155	(0.107)		2228	(56.9)	0.180	(0.124)	
**Education Level**					0.007					<0.0001
≤Elementary School	758	(28.2)	0.192	(0.126)	(0.0565)	2141	(54.6)	0.216	(0.129)	(0.1399)
Middle School	467	(17.4)	0.154	(0.103)		660	(16.8)	0.145	(0.098)	
High School	973	(36.3)	0.137	(0.095)		923	(23.6)	0.119	(0.089)	
≥College	486	(18.1)	0.125	(0.091)		195	(5.0)	0.101	(0.067)	
**Income Level**					0.017					0.207
Low	299	(11.1)	0.215	(0.112)	(0.1133)	741	(18.9)	0.235	(0.128)	(0.0838)
Middle-Low	318	(11.8)	0.213	(0.134)		498	(12.7)	0.201	(0.122)	
Middle-High	675	(25.1)	0.160	(0.106)		955	(24.4)	0.178	(0.117)	
High	1392	(51.9)	0.123	(0.089)		1725	(44.0)	0.142	(0.110)	
**Health insurance status**					0.001					<0.0001
Medical aid	135	(5.0)	0.231	(0.125)	(0.7661)	216	(5.5)	0.266	(0.155)	(0.7978)
National Health insurance	2549	(95.0)	0.149	(0.106)		3703	(94.5)	0.170	(0.118)	
**Economic activity**					<0.0001					<0.0001
No	1319	(49.1)	0.194	(0.126)	(0.8348)	2882	(73.5)	0.197	(0.131)	(0.6825)
Yes	1365	(50.9)	0.111	(0.064)		1037	(26.5)	0.117	(0.065)	
**Social activity**					<0.0001					<0.0001
No	569	(21.2)	0.233	(0.149)	(1.0034)	1114	(28.4)	0.249	(0.153)	(0.6825)
Yes	2115	(78.8)	0.132	(0.083)		2805	(71.6)	0.146	(0.092)	

FIS: Frailty Index Score/HLST: Healthy Lifestyle Score Trajectory/Effect sizes were calculated to quantify the magnitude of differences in Frailty Index Score across subgroups. Eta-squared (η^2^) was applied for variables with three or more categories (HLST, Age, Education Level, and Income Level), and Cohen’s d was applied for binary variables (Region, Economic Activity, Social Activity, and Health Insurance Status). For HLST, Cohen’s d was additionally calculated for each trajectory group relative to the Favorable HLST as the reference group. Effect size interpretation followed established criteria: for η^2^, values of <0.01, 0.01–0.06, 0.06–0.14, and ≥0.14 were classified as negligible, small, medium, and large, respectively; for Cohen’s d, values of <0.20, 0.20–0.50, 0.50–0.80, and ≥0.80 were classified as negligible, small, medium, and large, respectively.

**Table 2:** Adjusted effect between HLST and FIS by sex.

Variables	Male				Female			
	FIS							
	*B*	95% CI		*p*-Value	*B*	95% CI		*p*-Value
**HLST among males**								
Poor HLST	**0.018**	**(0.007, 0.029)**		**0.002**	N/A			
Moderate HLST	**0.008**	**(0.000, 0.016)**		**0.042**				
Severely Deteriorating HLST	**0.019**	**(0.005, 0.033)**		**0.010**				
Mildly Deteriorating HLST	0.009	(−0.004, 0.022)		0.181				
Favorable HLST	Ref							
**HLST among females**								
Poor HLST	N/A				**0.039**	**(0.024, 0.055)**		**<0.0001**
Moderate HLST					**0.022**	**(0.016, 0.028)**		**<0.0001**
Deteriorating HLST					**0.019**	**(0.010, 0.027)**		**<0.0001**
Improving HLST					0.014	(−0.002, 0.031)		0.091
Favorable HLST					Ref			
**Age**								
55–64	Ref				Ref			
65–74	0.009	(0.005, 0.013)		<0.0001	0.016	(0.012, 0.019)		<0.0001
≥75	0.036	(0.030, 0.042)		<0.0001	0.054	(0.048, 0.059)		<0.0001
**Region**								
Urban	Ref				Ref			
Rural	0.001	(−0.005, 0.007)		0.741	0.003	(−0.002, 0.008)		0.313
**Education Level**								
≤Elementary School	Ref				Ref			
Middle School	−0.017	(−0.026, −0.007)		0.001	−0.037	(−0.044, −0.030)		<0.0001
High School	−0.023	(−0.031, −0.015)		<0.0001	−0.049	(−0.056, −0.042)		<0.0001
≥College	−0.033	(−0.042, −0.024)		<0.0001	−0.057	(−0.068, −0.046)		<0.0001
**Income Level**								
Low	Ref				Ref			
Middle-Low	0.002	(−0.007, 0.011)		0.631	−0.007	(−0.012, −0.002)		0.011
Middle-High	−0.006	(−0.014, 0.003)		0.174	−0.010	(−0.015, −0.005)		0.000
High	−0.011	(−0.019, −0.002)		0.015	−0.008	(−0.014, −0.003)		0.004
**Health insurance status**								
Medical aid	Ref				Ref			
National Health insurance	0.033	(0.019, 0.046)		<0.0001	−0.036	(−0.046, −0.026)		<0.0001
**Economic activity**								
No	Ref				Ref			
Yes	0.033	(0.029, 0.037)		<0.0001	0.026	(0.022, 0.029)		<0.0001
**Social activity**								
No	Ref				Ref			
Yes	0.044	(0.039, 0.049)		<0.0001	0.039	(0.035, 0.042)		<0.0001
**Year of survey**								
2016	Ref				Ref			
2018	0.002	(−0.001, 0.006)		0.181	0.001	(−0.002, 0.003)		0.575
2020	−0.002	(−0.006, 0.001)		0.198	−0.004	(−0.007, −0.001)		0.009
2022	−0.011	(−0.015, −0.007)		<0.0001	−0.013	(−0.017, −0.010)		<0.0001
2024	0.007	(0.002, 0.012)		0.005	0.010	(0.006, 0.014)		<0.0001

Bold: *p*-value < 0.05. FIS: Frailty Index Score/HLST: Healthy Lifestyle Score Trajectory.

**Table 3:** Stratified analysis between HLST and FIS by sex and region.

Variables	Male			Variables	Female		
	FIS				FIS		
	HR	95% CI	*p*-Value		HR	95% CI	*p*-Value
	**Urban**				**Urban**		
**HLST among males**				**HLST among females**			
Poor HLST	0.005	(−0.010, 0.020)	0.523	Poor HLST	**0.045**	**(0.023, 0.067)**	**<0.0001**
Moderate HLST	0.008	(−0.004, 0.020)	0.178	Moderate HLST	**0.023**	**(0.014, 0.032)**	**<0.0001**
Severely Deteriorating HLST	0.011	(−0.007, 0.029)	0.244	Deteriorating HLST	**0.025**	**(0.012, 0.037)**	**<0.0001**
Mildly Deteriorating HLST	−0.003	(−0.021, 0.015)	0.776	Improving HLST	0.015	(−0.005, 0.034)	0.145
Favorable HLST	Ref			Favorable HLST	Ref		
	**Rural**				**Rural**		
**HLST among males**				**HLST among females**			
Poor HLST	**0.029**	**(0.013, 0.044)**	**0.000**	Poor HLST	**0.037**	**(0.016, 0.058)**	**0.001**
Moderate HLST	0.009	(−0.002, 0.019)	0.095	Moderate HLST	**0.022**	**(0.013, 0.030)**	**<0.0001**
Severely Deteriorating HLST	**0.026**	**(0.005, 0.047)**	**0.016**	Deteriorating HLST	**0.015**	**(0.004, 0.027)**	**0.009**
Mildly Deteriorating HLST	**0.018**	**(0.000, 0.036)**	**0.050**	Improving HLST	0.016	(−0.010, 0.042)	0.236
Favorable HLST	Ref			Favorable HLST	Ref		

All covariates were controlled. Bold: *p*-value < 0.05; FIS: Frailty Index Score/HLST: Healthy Lifestyle Score Trajectory.

## Data Availability

The data supporting the findings of this study are openly available at https://survey.keis.or.kr/eng/klosa/klosa01.jsp (accessed on 14 February 2026).

## Supplementary Materials

The following supporting information can be downloaded at: https://www.mdpi.com/article/10.3390/medicina62040766/s1, Table S1. Flowchart for sample selection; Table S2. Comparison between the analytic and excluded samples; Table S3. Best fitting model of trajectory class; Table S4. Average posterior probability of trajectory model; Table S5. Criteria for Frailty Index Measurement; Table S6. Study timeline.

## Abbreviations

The following abbreviations are used in this manuscript:

ADL	Activities of Daily Living
BMI	Body Mass Index
CES-D	Center for Epidemiological Studies Depression Scale
CI	Confidence Interval
FI	Frailty Index
FIS	Frailty Index Score
GBTM	Group-Based Trajectory Modeling
GEE	Generalized Estimating Equations
HLS	Healthy Lifestyle Score
HLST	Healthy Lifestyle Score Trajectory
IADL	Instrumental Activities of Daily Living
KloSA	Korean Longitudinal Study of Aging
PA	Physical Activity
SD	Standard Deviation
WHO	World Health Organization

## References

[1] Fried L.P., Tangen C.M., Walston J., Newman A.B., Hirsch C., Gottdiener J., Seeman T., Tracy R., Kop W.J., Burke G. (2001). Frailty in older adults: Evidence for a phenotype. J. Gerontol. A Biol. Sci. Med. Sci..

[2] Rockwood K., Mitnitski A. (2007). Frailty in relation to the accumulation of deficits. J. Gerontol. A Biol. Sci. Med. Sci..

[3] Xue Q.L. (2011). The frailty syndrome: Definition and natural history. Clin. Geriatr. Med..

[4] Cunha A.I.L., Veronese N., Borges S.d.M., Ricci N.A. (2019). Frailty as a predictor of adverse outcomes in hospitalized older adults: A systematic review and meta-analysis. Ageing Res. Rev..

[5] United Nations, Department of Economic and Social Affairs (2023). World Population Ageing 2023.

[6] Statistics Korea (2021). Population Projections for Korea (2020–2070).

[7] Kojima G., Iliffe S., Walters K. (2015). Smoking as a predictor of frailty: A systematic review. BMC Geriatr..

[8] Lafortune L., Martin S., Kelly S., Kuhn I., Remes O., Cowan A., Brayne C. (2016). Behavioural risk factors in mid-life associated with successful ageing, disability, dementia and frailty in later life. PLoS ONE.

[9] van Assen M.A., Helmink J.H., Gobbens R.J. (2022). Associations between lifestyle factors and multidimensional frailty: A cross-sectional study among community-dwelling older people. BMC Geriatr..

[10] Haapanen M.J., Mikkola T.M., Jylhävä J., Wasenius N.S., Kajantie E., Eriksson J.G., von Bonsdorff M.B. (2024). Lifestyle-related factors in late midlife as predictors of frailty. Age Ageing.

[11] Li Y., Schoufour J., Wang D.D., Dhana K., Pan A., Liu X., Song M., Liu G., Shin H.J., Sun Q. (2020). Healthy lifestyle and life expectancy free of cancer, cardiovascular disease, and type 2 diabetes. BMJ.

[12] Nagin D.S. (1999). Analyzing developmental trajectories: A semiparametric, group-based approach. Psychol. Methods.

[13] Gordon E.H., Peel N.M., Samanta M., Theou O., Howlett S.E., Hubbard R.E. (2017). Sex differences in frailty: A systematic review and meta-analysis. Exp. Gerontol..

[14] Gordon E.H., Hubbard R.E. (2019). Do sex differences in chronic disease underpin the sex-frailty paradox?. Mech. Ageing Dev..

[15] Collard R.M., Boter H., Schoevers R.A., Oude Voshaar R.C. (2012). Prevalence of frailty in community-dwelling older persons: A systematic review. J. Am. Geriatr. Soc..

[16] Yang J.M., Hwang J. (2025). Effect of healthy lifestyle score trajectory on all-cause mortality in the late middle-aged and older population: Finding from 17-year retrospective cohort study. Exp. Gerontol..

[17] Hoogendijk E.O., Rockwood K., Theou O., Armstrong J.J., Onwuteaka-Philipsen B.D., Deeg D.J.H., Huisman M. (2018). Tracking changes in frailty throughout later life: Results from a 17-year longitudinal study in the Netherlands. Age Ageing.

[18] Baek W., Min A. (2022). Frailty index and gender-specific mortality in Korean adults: Findings from the Korean Longitudinal Study of Aging (2006–2018). J. Adv. Nurs..

[19] Jung Y.J., Kim J., Jang Y.S., Park E.C. (2025). Retrospective observational study of the association between changes in physical activity and frailty in middle-aged and older adults: Evidence from the Korean Longitudinal Study of Aging (2006–2022). BMJ Open.

[20] McPhee J.S., French D.P., Jackson D., Nazroo J., Pendleton N., Degens H. (2016). Physical activity in older age: Perspectives for healthy ageing and frailty. Biogerontology.

[21] Hu Y., Sun C., Gu J., Huo X., Chen S., Zhao M., Li M. (2026). Association between healthy lifestyle index trajectories and functional disability in middle-aged and older adults: A longitudinal cohort study. Arch. Phys. Med. Rehabil..

[22] Wu Y., Peng H., Xu R., Hua Y., Zhang Y. (2025). Association of lifestyle modifications with frailty in older adults: A cross-sectional study using NHANES. J. Nutr. Health Aging..

[23] Howlett S.E., Rutenberg A.D., Rockwood K. (2021). The science of frailty: Sex differences. Mech. Ageing Dev..

[24] Zeidan R.S., McElroy T., Rathor L., Martenson M.S., Lin Y., Mankowski R.T. (2023). Sex differences in frailty among older adults. Mech. Ageing Dev..

[25] Ko Y., Choi K. (2017). Prevalence of frailty and associated factors in Korean older women: The KLoSA study. J. Women Aging.

[26] Searle S.D., Mitnitski A., Gahbauer E.A., Gill T.M., Rockwood K. (2008). A standard procedure for creating a frailty index. BMC Geriatr..

[27] Zhong W.F., Wang X.M., Song W.Q., Chen H., Xie J.H., Yan H., Wang J.J., Lv Y.B., Li Z.H., Shi X.M. (2025). Three-year lifestyle changes, genetic risk, and risk of frailty among older adults: A national community-based cohort study. Eur. Geriatr. Med..

[28] Yang M., Liu Y., Matsumoto M., Jiao D., Zhu Z., Li X., Cui M., Zhang J., Qian M., Huang L. (2025). Association of healthy lifestyle patterns with changes in physical frailty and subjective cognitive function among community-dwelling older adults: A 3-year longitudinal study. BMC Geriatr..

[29] Jang I.Y., Jung H.W., Lee C.K., Lee Y.S., Kim K.-I., Kim K.W., Oh H., Ji M.Y., Lee E., Kim D.H. (2016). Rural and urban disparities in frailty and aging-related health conditions in Korea. J. Am. Geriatr. Soc..

[30] Seo Y., Kim M., Shim H., Won C.W. (2021). Differences in the association of neighborhood environment with physical frailty between urban and rural older adults: The Korean Frailty and Aging Cohort Study (KFACS). J. Am. Med. Dir. Assoc..

