# Supplementary material for: The Association Between Healthy Lifestyle Score Trajectory and Frailty in Middle-Aged and Older Adults in Korea: Findings from the Korean Longitudinal Study of Aging (2006–2024)
Source: Medicina (Kaunas). 2026 Apr 15;62(4):766. doi: 10.3390/medicina62040766 (PMC13117357; doi:10.3390/medicina62040766)

Supplementary Table S1. Flowchart for sample selection

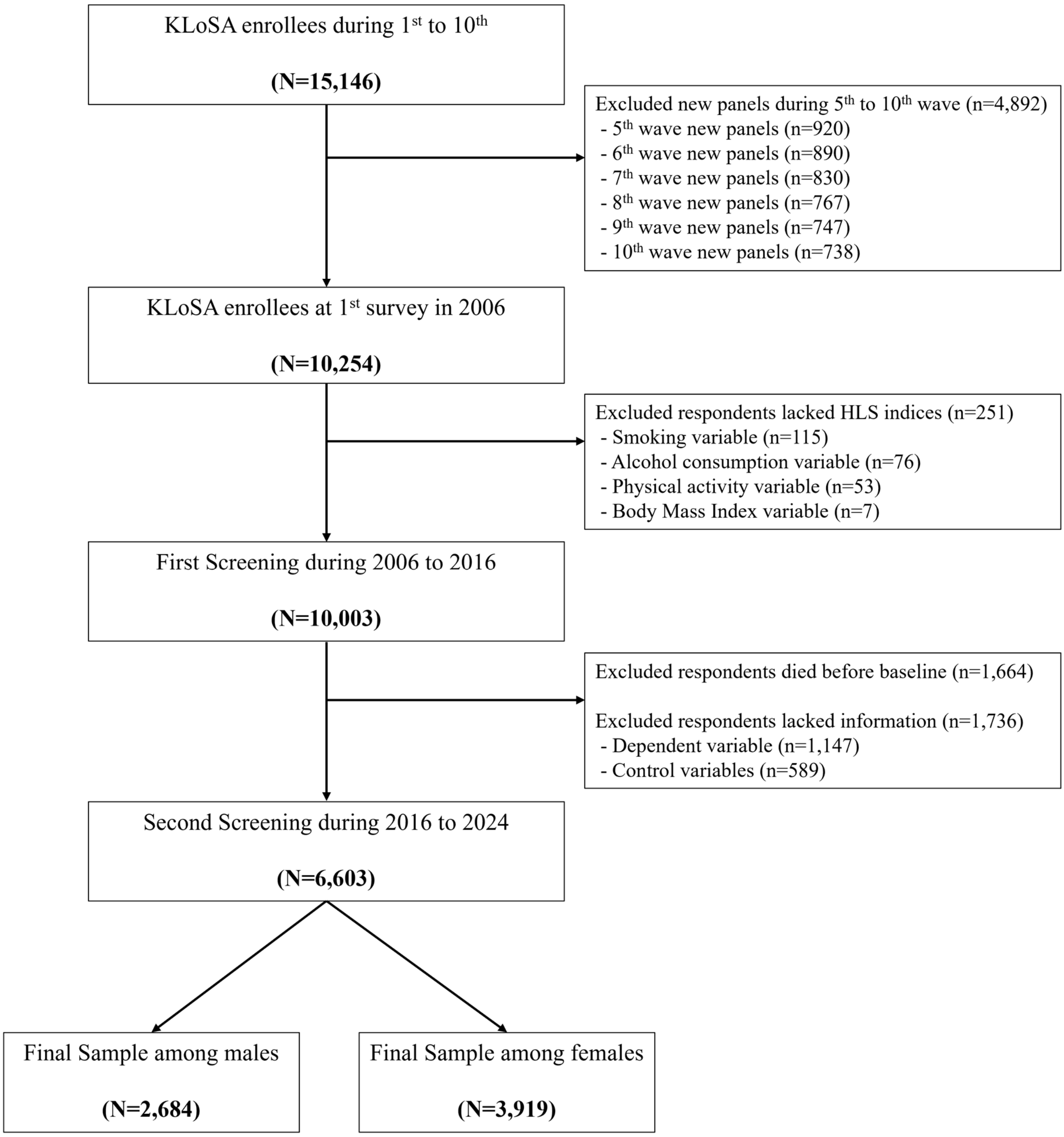

Supplementary Table S2. Comparison between the analytic and excluded samples

Male			
**Variables**	**Non-excluded sample Proportion (%)**	**Excluded sample Proportion (%)**	**Diff.**
**Age**			
**55-64**	**35.0**	**19.92**	*
**65-74**	**31.94**	**30.65**	*
**≥75**	**32.3**	**49.43**	*
**Region**			
Urban	42.92	37.55	
Rural	57.08	62.45	
**Education Level**			
≤ Elementary School	43.90	46.36	
Middle School	17.07	22.61	
High School	22.61	20.31	
≥ College	10.31	10.73	
**Income Level**			
**Low**	**15.75**	**19.54**	*
**Middle-Low**	**12.36**	**16.48**	*
**Middle-High**	**24.69**	**27.20**	*
**High**	**47.21**	**36.78**	*
**Health insurance status**			
Medical aid	5.32	6.90	
National Health insurance	94.68	93.10	
**Economic activity**			
No	64.32	69.35	
Yes	35.68	30.65	
**Social activity**			
No	**74.51**	**62.84**	*
Yes	**24.49**	**37.16**	*
**Total participants**	**6,603**	**1,046**	

* Differences between analytic sample and excluded sample statistically significant, $p < 0.05$.

Supplementary Table S3. Best fitting model of trajectory class

Groups for HLST among males				
**Number of groups**	**Null model**	**AIC**	**BIC²**	**BIC³**
2	1	-28,396.23	-28,429.55	-28,435.09
3	2	-27,348.92	-27,398.90	-27,407.21
4	3	-26,917.46	-27,984.11	-27,995.19
**5 (Elbow point)**	**4**	**-26,706.77**	**-26,790.07**	**-26,803.92**
6	5	-26,612.93	-26,688.90	-26,705.52
7	6	-26,558.39	-26,647.01	-26,666.40

BIC²= Bayesian information criterion (for the total number of participants) / N=2,684
BIC³= Bayesian information criterion (for the total number of observations) / N=10,723

Groups for HLST among females				
**Number of groups**	**Null model**	**AIC**	**BIC²**	**BIC³**
2	1	-40,355.13	-40,381.83	-40,387.73
3	2	-39,206.19	-39,246.24	-39,255.09
4	3	-38,170.33	-38,223.73	-38,235.53
**5 (Elbow point)**	**4**	**-37,637.43**	**-37,737.18**	**-37,718.93**
6	5	-37,532.47	-37,612.57	-37,030.28
7	6	-37,419.70	-37,513.15	-37,533.80

*HLST: Healthy Lifestyle Score Trajectory
BIC²= Bayesian information criterion (for the total number of participants) / N=3,919
BIC³= Bayesian information criterion (for the total number of observations) / N=17,138

Supplementary Table S4. Average posterior probability of trajectory model

	Groups for HLST among males				
	(1) Poor HLST	(2) Moderate HLST	(3) Severely Deteriorating HLST	(4) Mildly Deteriorating HLST	(5) Favorable HLST
Average Posterior probability (%)	91.3	94.7	96.2	92.8	98.1
	**Groups for HLST among females**				
	(1) Poor HLST	(2) Moderate HLST	(3) Deteriorating HLST	(4) Improving HLST	(5) Favorable HLST
	90.6	93.5	97.4	95.9	91.2

*HLST: Healthy Lifestyle Score Trajectory

Supplementary Table S5. Criteria for Frailty Index Measurement

Variables	Score
**1. Self-rating of health**	Excellent (0)<br>Very good (0.25)<br>Good (0.5)<br>Fair (0.75)<br>Poor (1)
**2. Physical condition**	
(1) Impaired vision	Excellent (0)<br>Very good (0.25)<br>Good (0.5)<br>Fair (0.75)<br>Poor (1)
(2) Impaired hearing	Excellent (0)<br>Very good (0.25)<br>Good (0.5)<br>Fair (0.75)<br>Poor (1)
(3) Sleep disturbance	Rarely or never (0)<br>Some of the time (0.33)<br>Occasionally (0.66)<br>All of the time (1)
(4) Weight loss	No (0)<br>Yes (1)
(5) Limitation in usual activities due to health problem	Strongly disagree (0)<br>Disagree (0.33)<br>Agree (0.66)<br>Strongly agree (1)
(6) BMI	>18.5 kg/m^2 (0)<br>≤18.5 kg/m^2 (1)
(7) Grip strength	Male: >28.6, Female: >16.4 (0)<br>Male: ≤28.6, Female: ≤16.4 (1)
**3. Activities of daily living**	
(1) Help dressing	Able (0)<br>With help (0.5)<br>Unable (1)
(2) Help personal hygiene	Able (0)<br>With help (0.5)<br>Unable (1)
(3) Help bathing	Able (0)<br>With help (0.5)<br>Unable (1)
(4) Help getting in/out of bed	Able (0)<br>With help (0.5)<br>Unable (1)
**4. Instrumental activities of daily living**	
(1) Help grooming	Able (0)<br>With help (0.5)<br>Unable (1)
(2) Help with housework	Able (0)<br>With help (0.5)<br>Unable (1)
(3) Help with meal preparations	Able (0)<br>With help (0.5)<br>Unable (1)

(4) Help with laundry	Able (0)<br>With help (0.5)<br>Unable (1)
(5) Help walking around house	Able (0)<br>With help (0.5)<br>Unable (1)
(6) Help with using transportation	Able (0)<br>With help (0.5)<br>Unable (1)
(7) Help shopping	Able (0)<br>With help (0.5)<br>Unable (1)
(8) Help with finances	Able (0)<br>With help (0.5)<br>Unable (1)
(9) Help phone use	Able (0)<br>With help (0.5)<br>Unable (1)
(10) Help taking medication	Able (0)<br>With help (0.5)<br>Unable (1)
**5. Chronic conditions**	
(1) Hypertension	No (0)<br>Yes (1)
(2) Diabetes	No (0)<br>Yes (1)
(3) Chronic lung disease	No (0)<br>Yes (1)
(4) Heart disease	No (0)<br>Yes (1)
(5) Stroke	No (0)<br>Transient ischemic attack (0.5)<br>Yes (1)
(6) Arthritis	No (0)<br>Yes (1)
(7) Urinary incontinence	No (0)<br>Yes (1)
(8) Regular prescribed medications	No (0)<br>Yes (1)
**6. Mental status**	
(1) I had trouble keeping my mind on what I was doing	Rarely or never (0)<br>Some of the time (0.33)<br>Occasionally (0.66)<br>All of the time (1)
(2) I felt everything I did was an effort	Rarely or never (0)<br>Some of the time (0.33)<br>Occasionally (0.66)<br>All of the time (1)
(3) I felt lonely	Rarely or never (0)<br>Some of the time (0.33)<br>Occasionally (0.66)<br>All of the time (1)
(4) I could not get "going."	Rarely or never (0)<br>Some of the time (0.33)<br>Occasionally (0.66)<br>All of the time (1)

Supplementary Table S6. Study timeline

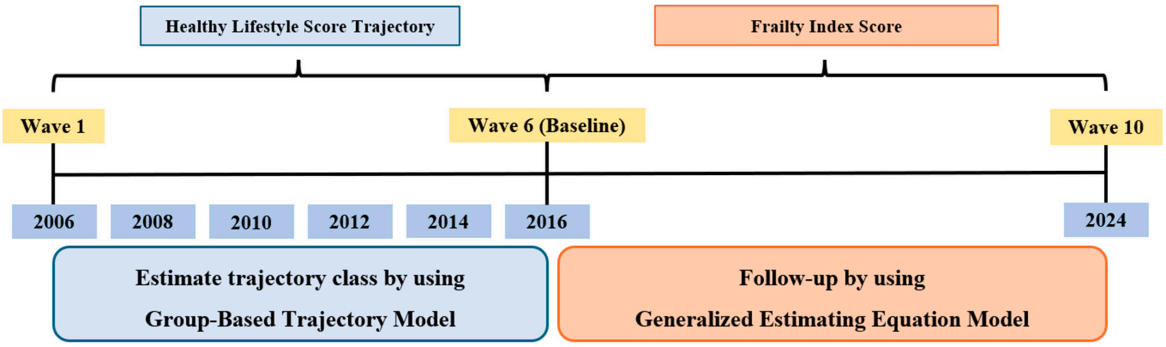

Supplement: Supplementary file 1 [file medicina-62-00766-s001.zip › medicina-4243329-supplementary.pdf]
